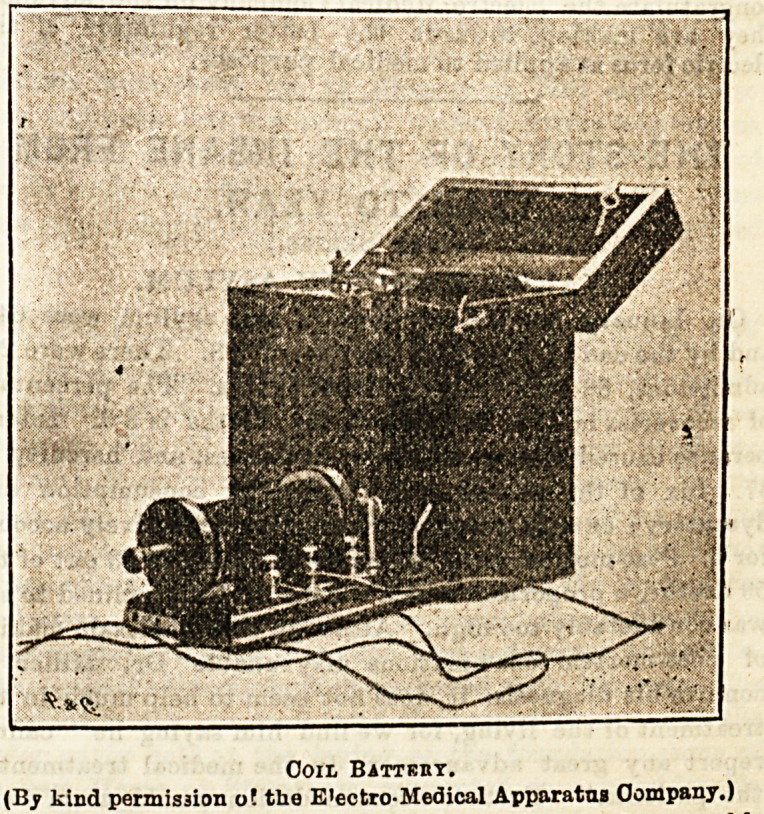# L.K. Batteries

**Published:** 1893-05-13

**Authors:** 


### PRACTICAL DEPARTMENTS.

### L.K. BATTERIES.

The Electro-Medical Apparatus Company, Trafalgar Chambers, 36, St. Martin's Lane, under the management of Messrs. H. N. Lawrence, M.I.E.E., and Alan Kirk, can provide for every application of medical electricity at their

COIL BATTERY.
(By kind permission of the Electro-Medical Apparatus Company.)

chambers in St. Martin's Lane, and they also supply portable batteries and other apparatus on hire by the month. Nurses are not infrequently called upon to administer electricity, and sometimes have also to provide the battery for the purpose. In purchasing or hiring portable batteries it is very necessary to obtain such as are suitable, reliable, and simple of construction. Some makers rely upon one particular feature of their apparatus, and some upon another, but the Electro-Medical Apparatus Company would seem to have combined most, if not all, the chief essentials in their L.K. Batteries. The cells in all these batteries contain no acid or other fluid to spill about, so that they may with safety be placed in any position. They possess a high E M.F., and greater capacity than most of the fluid cells even of larger size. The batteries themselves are arranged in the simplest possible manner. The continuous current battery of 20 dry cells is contained in a highly polished walnut case, with plated metal work, and may be fitted with terminals or with an ingenious new form of double cell collector which enables cells to be used in any part of the series, so that the current may be drawn fairly from all. The coil battery is fitted with a dry cell of great capacity (as shown in the illustration) and with terminals for both primary and secondary wires. It is a handsome battery and simplicity itself, for opening the box puts the coil into action and shutting the box stops it. The vibrations of the hammer are smooth and regular, while the gradation of the current is very even and complete. It has been very difficult hitherto satisfactorily to regulate the electric force, but its management is now becoming daily better understood, and there is certainly much to be hoped for in the future from its use as a therapeutic agent.

The above mentioned company have recently brought out a new and convenient rheostat for use with small currents. It has a wide range varying from ten to a million ohms, and is capable of very minute graduation. The instrument is extremely simple, and is the safest we have seen, especially for medical use, for there is no risk of breakdown or dangerous heating, and the advantages of so wide a range in a cheap instrument are very considerable. The only drawback is occasionally having to remoisten the sponge, but that may be done in a few seconds. This rheostat is likely largely to supersede cell collectors in medical work, for by its adoption all the cells of a battery must be used fairly, inasmuch as a small portion of the total current passed through the circuit must come from each. Further, this rheostat enables a more gradual and smooth regulation of the current than can be obtained from any form of cell collector. We congratulate the Electro-Medical Company on the advances they are making towards the better regulation of the electric force as applied to medical purposes.

## Figures and Tables

**Figure f1:**